# The first experimental study of transference work–in teenagers (FEST–IT): a multicentre, observer- and patient-blind, randomised controlled component study

**DOI:** 10.1186/s12888-021-03055-y

**Published:** 2021-02-17

**Authors:** Randi Ulberg, Benjamin Hummelen, Anne Grete Hersoug, Nick Midgley, Per Andreas Høglend, Hanne-Sofie Johnsen Dahl

**Affiliations:** 1 Division of Mental Health and Addiction, Institute of Clinical Medicine, University of Oslo, P.O. box 1171, 0318, Blindern, Oslo, Norway; 2 Vestfold Hospital Trust, Division of Mental Health, Research Unit, P.O. box 2169, 3125 Tønsberg, Norway; 3 Department of Psychiatry, Diakonhjemmet Hospital, Box 85 Vinderen 0319, Oslo, Norway; 4 Division of Mental Health and Addiction, Oslo University Hospital, P.O. box 4959, N-0424, Nydalen, Oslo, Norway; 5 University College London (UCL) and Anna Freud National Centre for Children and Families, London, UK; 6 Department of Psychology, Faculty of Social Sciences, University of Oslo, Postboks 1094 Blindern, 0317 Oslo, Norway

**Keywords:** Psychodynamic, Psychoanalytic, Youth psychotherapy, Transference, Major Depressive Disorder

## Abstract

**Background:**

Little is known about the influence on outcome of exploration of the patient-therapist relationship (that is, transference work) in psychoanalytic psychotherapy. We hypothesized that depressed adolescents would have better long-term effects from psychoanalytic psychotherapy with than without transference work.

**Methods:**

Depressed adolescent (16 to 18 years) were recruited in health authority funded out-patient clinics in Oslo and Vestfold County, Norway. They were randomized to 28 weeks of treatment with psychoanalytic psychotherapy with or without transference work. Change was assessed using linear-mixed models. The primary outcome measure was the Psychodynamic Functioning Scale (pre- post-, and 1-year post-treatment). Level of depression was measured at the same time points and during therapy (week 12, and 20).

**Results:**

69 adolescents were treated with (*N* = 39) or without (*N* = 31) transference work. The mean number of sessions was 18.6 (SD = 8,6) in the transference work group and 18.0 (SD = 10.9) in the non-transference work group.

Both groups showed large and significant improvement on Psychodynamic Functioning Scale during the whole study period. The difference between the two groups was not significant during the treatment period (95% CI −.79 to 1.2, *p* = .674, F = .18), or from post-treatment to one-year follow-up (95% CI −.13 to .96; *p* = .134; F = 2.3). For the secondary outcome measures the transference work group had significantly better outcomes from 12 weeks in treatment to one-year follow-up (Beck Depression Inventory, 95% CI − 1.7 to −.14, *p* = .022; Montgomery and Åsberg Depression Rating Scale, 95% CI − 1.6 to −.23, *p* = .009).

**Conclusion:**

The findings suggest that exploration of the adolescents’ relations to the therapist amplify the effects of short-term psychoanalytic psychotherapy on their depressive symptoms for adolescents with a Major Depressive Disorder.

**Trial registration:**

ClinicalTrials.gov. Id: NCT01531101. Registered 8 February 2012.

**Supplementary Information:**

The online version contains supplementary material available at 10.1186/s12888-021-03055-y.

## Background

Mental health problems, such as anxiety and depression, are estimated to affect 30% of youth worldwide. 50% of lifetime diagnosable mental health disorders start by the age of 14, and this number increases to 75% by the age of 24. There is a need for a more comprehensive evidence base for treatment of young people with mental health problems, including depression [1].

Empirical evidence supports treatment with psychoanalytic psychotherapy in adolescents [2–4]. Short-term psychoanalytic psychotherapy has been shown to promote improvement in depressed young individuals [4, 5]. The study “Improving mood with psychoanalytic and cognitive therapies” (IMPACT) is the most extensive trial in the field of youth depression psychotherapy [4]. In IMPACT, three manualized psychotherapy modes were compared. Short-term psychoanalytic therapy, cognitive behavioural therapy (CBT), and a brief psychosocial intervention were all found equal in terms of clinical- and cost-effectiveness. The authors reported no evidence for the superiority of any of the three treatment modes in maintenance of reduced depression symptoms approximately 12 months after treatment [4].

The IMPACT trial was not designed to establish whether the comparable outcomes were due to common factors, or whether there were distinctive features which led to comparable outcomes. There is probably an array of active ingredients in psychotherapy. However, there is a need for more knowledge on how specific psychotherapeutic techniques in the different treatment modes influence outcome over time [3, 5, 6]. The young person’s experience of the therapist and the therapy is assumed to be of importance for treatment outcome [7–9]. In psychoanalytic psychotherapy the in-session exploration of the patient - therapist relationship (that is, transference work), is a key component. However one Randomized clinical trial (RCT) with adult patients, the First Experimental Study of Transference–Interpretations (FEST), found no main effects of this specific technique [10, 11]. The present study, FEST-IT, is an adjusted replication of FEST, including adolescents with Major Depressive Disorder (MDD). The treatment is according to the treatment manual in the short-term psychoanalytic arm of IMPACT [12]. One ingredient described in the manual, the transference work, is manipulated in the two treatments in FEST-IT.

The purposes of psychoanalytic psychotherapy for MDD are reduction of depressive symptoms, but also to foster dynamic change in the young person. Positive dynamic change includes a broad spectre of areas; i.e. improved relations, increased insight in one’s own reactions, enhanced tolerance for affects, and improved capacity to solve upcoming problems in life. Transference work are thought to promote dynamic change, a phenomenon which is challenging to measure. Psychodynamic Functioning Scales (PFS) [13] is a measure developed to capture evaluator-rated dynamic change. PFS has shown promising features when it comes to capture statistically and clinically significant changes in adolescents [14]. The patient’s functioning during the last three months is rated based on a semi-structured dynamic interview [15]. Five sub scales relevant for rating adolescents are: Quality of Family Relations, Quality of Friendships, Tolerance for Affects, Insight, and Problem-Solving Capacity.

Building on the design of the two previous clinical trials, i.e. IMPACT and FEST, the FEST-IT trial aims to assess the effects of transference work in adolescent psychotherapy for young people with MDD. As in IMPACT, the included patients were youth with MDD. As in FEST, the patients were randomized to short-term psychoanalytic therapy with or without transference work. The primary hypothesis in FEST-IT was that the transference work group would have a more favourable course than the non-transference work group over time. That is, a significant improvement during the whole study period, on the PFS, and on patient rated depression measure Beck Depression Inventory (BDI) as well as the clinician rated Montgomery Åsberg Rating Scale (MADRS). Adolescence is a time to explore and develop relational and emotional skills as well as social competency. When a depressed one withdraws from friends and family, talking about the ongoing relationship with the therapist in the here and now invites the adolescent to practice relational and emotional skills. The aim of the study was to decide whether depressed adolescents improve significantly more from short-term psychoanalytic psychotherapy with than without transference work [16], up to 1-year follow-up, both in terms of overall functioning (PFS), as well as depression symptoms (BDI and MADRS).

## Methods

### Study design and participants

Experimental Study of Transference Work-In Teenagers (FEST-IT) is a multicentre, observer- and patient-blind, randomised controlled component trial. The patients were included from two areas in Norway: the mainly urban areas in and around Oslo of about 1 million people, and mixed urban and rural areas in Vestfold County containing about 250,000 people. The patients were treated in outpatient clinics.

Adolescents aged 16 to 18 years, with current unipolar MDD according to Diagnostic and Statistical Manual of Mental Disorders, Fourth Edition (DSM-IV; American Psychiatric Association, 2000), were included. Adolescents with generalized learning difficulties, pervasive developmental disorder, psychosis, or substance addiction were excluded. Comorbidity was expected to be frequent. Axis I and II diagnosis were based on the Mini International Neuropsychiatric Interview (M.I.N.I.) [17] and Structured Interview for DSM-IV Personality (SIDP-IV) [18]. Written consent was obtained from all patients.

### Randomisation and masking

The randomisation was stratified. The allocation to treatment of patients admitted to the trial was achieved by setting up clusters of four patients for each of the 12 therapists and using lottery to allocate patients to one of the two treatment groups. For each of four patients randomised to each therapist, two patients were treated with transference work and two without. A randomisation officer with no other connection with the evaluators, therapists, study coordinator, or researchers did the randomisation. Only the therapist was aware of the randomisation, which was concealed from patients and evaluators. The randomisation key was kept by the officer and broken when the last patient had finalized the study treatment and had been evaluated at 1-year follow up.

### Procedure

The 12 therapists treated patients in both treatment groups. The treatment manual developed by the IMPACT research group and used in the short-term psychoanalytic arm of IMPACT [12], was used. The patients were offered 28 sessions. The therapy mode emphasizes general psychoanalytic treatment principles, i.e. interventions exploring the young person’s relationships to others, thoughts and feelings that might be avoided, as well as pointing to recurring patterns in both feelings, thoughts, and behaviour. The therapists are receptive to what the patient finds important to talk about and do not define the focus for the sessions. The patients were randomised to short-term psychoanalytic psychotherapy with either transference work or no transference work. Transference work is explained in more detail in the Supplement, Supplementary file 1: Appendix I. In the transference work group, the therapists encouraged the patients to explore their feelings and thoughts about the therapist and the therapy, as well as repetitive patterns of reactions and actions emerging during the sessions in relationship to the therapist. These interventions were offered to a moderate level (i.e. 1–3 times per session).

The therapists were experienced psychologists and psychiatrists. They had a minimum of two years of psychoanalytic training and were also trained through a one-year course with two full day seminars and monthly half day seminars based on the treatment manual [12]. During the preparation course, the focus was on the differences in the techniques when offering short-term psychoanalytic psychotherapy with or without transference work. To maintain the quality of the therapies and adherence to the manual, peer supervision groups were offered throughout the study period. This also was to ensure that the therapy mode in each therapy group was delivered. Certified supervisors in psychoanalytic psychotherapy managed the continuous training. The Principal Investigator and the study coordinator were available for the therapists at any time. The average number of attended sessions were 18.6 (SD = 8.6) in the transference work group and 18.0 (SD = 10.9) in the non-transference work group. The therapists’ use of the specific transference techniques differentiated significantly between the treatment groups. The level of the transference interventions were measured on a Likert scale 0–4; 2.2 (SD 1.47) in the transference work group and .52 (SD .78) in the non-transference work group (df 52.5, t = − 5.5 *p* < 0,0006). Intra Class Correlation among two raters (single measure) was .89 (CI 95% .75 to .96, *p* = .000).

### Outcomes

The primary outcome measure was the Psychodynamic Functioning Scales (PFS). The five PFS-sub scales Quality of Family Relations, Quality of Friendships, Tolerance for Affects, Insight and Problem-Solving Capacity were used. The patients were rated with PFS by three raters blinded for randomisation at pre- and post-treatment and at 1-year follow-up. To measure the level of depression, the patients filled in a self-report scale, the BDI [19]. The MADRS [20] were rated by the therapists during therapy and by independent and blind raters at pre-, post-, and 1 year follow up. FEST-IT is a multicentre trial. Evaluators interviewed the patients and rated at pre-, post-, and 1-year follow-up. In addition to the evaluators, two experienced psychoanalysts rated all audio-taped interviews. To avoid skewness in ratings to develop, all raters were trained in scoring the PFS and MADRS and met for regular reliability ratings during the study period. The average of the evaluator ratings and the two experienced psychoanalysts’ ratings of each patient interview on each time point using PFS, were used in the statistical analyses. Intra Class Correlation (ICC) for PFS was .82 (CI 0.73–0.91) [15]. MADRS was rated by one independent and blinded rater and the therapist in 30% of the cases. ICC for MADRS single measure was .78 (Cl 0.58–0.9). During therapy MADRS was rated by the therapist.

### Statistical analyses

The Supplement, Supplementary file 1: Appendix II details the statistical analysis.

Based on standard t-test comparison of the two groups, 100 patients in this study would reveal a moderate effect size (0.55), with a significance level of 0.05 and a power of 0.80. However, only 69 patients were finally included in the study. With 39 patients in the transference work group and 30 patients in the non-transference work group, power is reduced to .61. An one-sided t-test gives a power of .72, which implies that the risk of not rejecting the 0-hypothesis, i.e., it is a 28% chance that no difference in treatment outcome with respect to PFS, is not true.

#### Linear mixed models analyses

The mixed models procedure in SPSS (version 26) was used to investigate differences in clinical change across the two treatment groups for PFS, BDI, and MADRS (dependent variables). The components “time” and “time*treatment” were entered as independent variables/covariates in the model. “Treatment” is a dummy variable indicating group membership, i.e., “transference treatment group” versus “non-transference treatment group”. The time variable was coded by integers and each integer represents approximately 10 weeks. For PFS, time was coded as 0–3-8, representing the three points in time when PFS was measured, i.e., pre-treatment, post-treatment, and one-year follow-up. For BDI and MADRS, time was coded as 0–1–2-3-8, representing five time points, i.e., pre-treatment, 12 weeks, 20 weeks, post-treatment, and one-year follow-up.. The interaction component “time*treatment” was included to test differential change rates across the two treatment groups. Log likelihood estimation (LLH) and Akaike Information Criterion (AIC) were used to evaluate model fit. Model parameters were estimated by maximum likelihood estimation and models were compared by log likelihood ratio tests.

The analyses were conducted in three steps (see online supplement for details). The first step aimed at modelling the mean response over time for the entire sample. The second step addressed the question whether there was differential change for the two treatment groups. The third step aimed at comparing differences in change rates across the two groups for different time periods within the same model, i.e., the “linear spline model”. For PFS, the knot was placed at post-treatment in the linear spline model, which implies that the first time period was from pre-treatment to post-treatment and the second period from post-treatment to one-year follow-up. For BDI and MADRS, the knot was situated at 12 weeks in treatment, which means that the first time period was from pre-treatment to 12 weeks in treatment and the second period from 12 weeks to one-year follow-up. The choice of the time periods was based on visual inspection of the scatterplots including interpolation lines through mean values for each group. As part of the sensitivity analyses, the three steps were reconducted by a competing linear spline model, i.e., a model with random intercepts and random slopes at the individual level, with variance component covariance matrix for the random effects (the “random effects model”).

Differences in the frequency of missing data across treatment groups were analysed be chi-square statistics, and differences in mean values of treatment outcomes were analysed by a series of ANOVAs including Tukey post hoc tests. On basis of these analyses, it was concluded that the missing data could be treated as “missing completely at random” in the current study.

The analyses were performed by an independent researcher overseen by one senior researcher at the University of Oslo and one statistician from research service at Oslo University Hospital. All 69 patients recruited from outpatient clinics included in the study were included in the intention to treat analyses. The Central Norway Regional Ethics Health Committee approved the study protocol (https://www.med.uio.no/klinmed/english/research/projects/fest-it/pdf/fest-it_protocol.pdf) (REK: 2011/1424 FEST-IT). FEST-IT is registered in ClinicalTrials.gov: NCT01531101.

### Role of the funding source

The funder of the study had no role in study design, data collection, data analysis, data interpretation, or writing of the report. The corresponding author had full access to all the data in the study and had final responsibility for the decision to submit for publication.

## Results

**Fig. 1:**
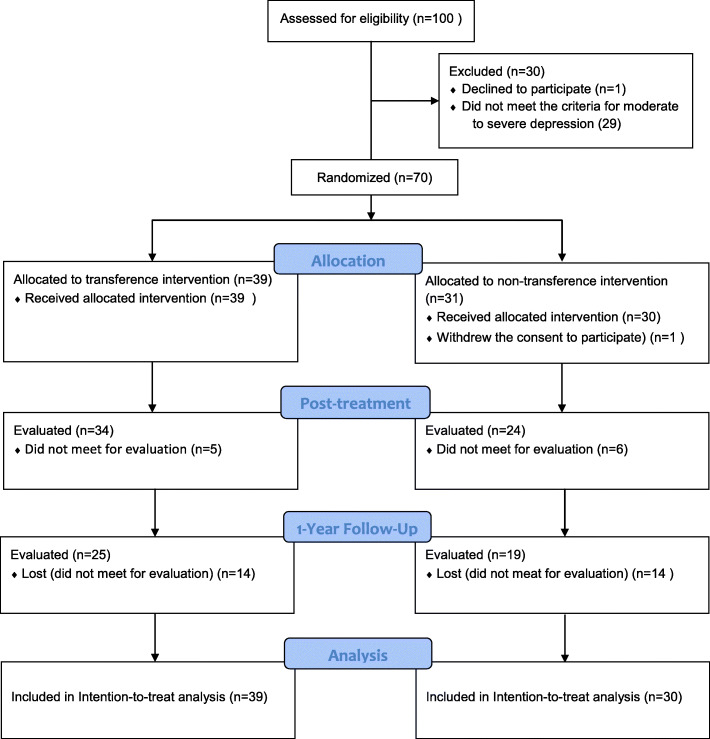
Trial profile. The primary hypothesis was analyzed in 69 patients. 16 patients had missing PFS at one occasion. 16 patients had missing BDI at one occasion. 11 patients had missing MADRS at one occasion. One patient in the non-transference group withdrew consent before starting treatment

Between February 2012 and September 2017, 70 patients were randomly assigned to psychoanalytic therapy with (*N* = 39) or without (*N* = 31) transference work. 1 patient withdrew from the study. Data from 69 patients were included in the intention-to-treat analyses (Please see the flow diagram, Fig. 1).

No significant differences were observed between the transference work group and the comparison group on the pre-treatment variables (Table 1).

**Table 1:** Pre-treatment characteristics in 69 adolescents receiving 28 weeks of psychoanalytical psychotherapy with or without transference work

	Transference work group (n = 39)		Non-transference work group (***n*** = 30)		Effect sizes
	*N*	%	*N*	%	
Gender					
Female	33	84.6	24	80.0	
Male	6	15.4	6	20.0	x^2^ = .25, *p* = .62
Housing situation					
Both parents	15	38.5	14	46.6	
One parent or commute between two parents	22	56.4	12	40.0	x^2^ = 2.5, *p* = .29
Other	2	5.1	3	10.0	
Missing	–	–	1	3.4	
Diagnostics (M.I.N.I.)					
Recurrent depression	15	38.5	9	30.0	x^2^ = .139, *p* = .71
Suicide risk (moderate to high)	6	15.4	4	13.3	Χ^2^ = .06, *p* = .81
Prevalence of one or more comorbid diagnoses	18	46.2	16	53.3	x^2^ = .35, *p* = .55
	Mean	(SD)	Mean	(SD)	
Age	17.30	(0.7)	17.31	(0.7)	t(66) = 0.64, *p* = .95
Personality diagnostics					
PD criteria as measured with SIDP-IV	13.5	(9.0)	12.4	(7.8)	t(65) = −0,52, *p* = .60

There was little use of medication among the patients. One patient used antidepressant medication at the beginning of therapy. One other at the end of therapy, however, not the same patient. One patient was taking antipsychotics throughout the study period. One patient at pre-treatment and 4 patients at post-treatment were taking sleeping medicine [14].

### Primary analyses of Outcome Variables.

Descriptive statistics over time for the three outcome variables are presented in Table 2.

**Table 2:** Outcome measures over time in 69 adolescents receiving short-term psychoanalytic psychotherapy with or without transference work

	Transference work group			Non-transference work group			Df	t	p
	N	Mean	(SD)	N	Mean	(SD)			
**Primary outcome measures**									
PFS (Psychodynamic Functioning Scales)									
Pre-treatment	39	61.07	(5.1)	30	61.24	(4.7)	67	.14	.89
Post-treatment	34	66.94	(6.2)	24	66.79	(6.3)	56	−.09	.93
One-year follow-up	25	71.02	(5.3)	19	68.48	(6.5)	42	−1.42	.16
**Depression measures**									
MADRS (Montgomery and Åsberg Depression Rating Scale)									
Pre-treatment	39	21.67	(6.1)	29	24.14	(6.0)	66	1.67	.10
12 weeks	27	14.33	(7.6)	19	13.58	(7.3)	44	−.34	.74
20 weeks	21	13.62	(5.9)	15	13.73	(7.4)	34	.52	.96
Post-treatment	33	12.76	(7.7)	25	11.90	(7.5)	56	−.43	.67
One-year follow-up	23	7.09	(7.0)	20	12.28	(8.0)	41	2.26	.03
BDI (Becks Depression Inventory)									
Pre-treatment	35	28.24	(9.7)	28	29.10	(8.4)	61	.39	.71
12 weeks	28	21.66	(11.7)	19	20.38	(11.7)	45	−.37	.71
20 weeks	21	19.04	(12.4)	15	20.57	(13.2)	34	.36	.72
Post-treatment	34	15.45	(11.6)	24	17.21	(14.3)	56	.52	.61
One-year follow-up	26	8.42	(10.9)	20	14.75	(11.9)	44	1.88	.07

There was a significant change for PFS from pre-treatment to post-treatment for the entire sample. Inspecting the first two rows of Table 3, it can be seen that for every 10 weeks, PFS increased 1.7 points for the non-transference work group (*p* = .000) and 1.91 points (1.7 + .21) for the transference work group (a non-significant difference; *p* = .674). From post-treatment to one-year follow-up, the increase of PFS was not significant for either groups (*p* = .083 for time, i.e., the non-transference work group, and *p* = .134 for time*treatment). The non-significant interactions between time and treatment indicate that the change rates did not differ significantly across the two treatment groups. However, in the linear model for PFS, the interaction between time and treatment was significant at the alpha = .05 level (F = 4.1, *p* = .048). In this model, the non-transference work group increased .75 for every ten weeks from pre-treatment to one-year follow-up whereas the transference work group increased 1.11 points for every ten weeks. The fit of this model was significantly poorer than the fit of the spline model (see online supplement for details).

**Table 3:** Results from linear spline models for Psychodynamic Functioning Scales, Beck Depression Inventory, and Montgomery and Åsberg Depression Rating Scale

Dependent variable	Estimate	SE	CI (95%)	Df	F-value	t	p
**Psychodynamic functioning scale**							
*From pre- to post-treatment*							
Time ^a^	1.7	.39	.92 to 2.5	62.0	46.5	4.3	.000
Time x treatment ^b^	.21	.50	−.79 to 1.2	58.1	.18	.67	.674
*Post-treatment to one-year follow-up*							
Time ^a^	.37	.21	−.05 to .78	47.3	17.8	1.8	.083
Time x treatment ^b^	.41	.27	- .13 to .96	47.0	2.3	1.5	.134
**Beck Depression Inventory**							
*From pre-treatment to 12 weeks*							
Time ^a^	−10.1	2.0	−14.0 to −6.1	65.1	43.8	−5.1	.000
Time x treatment ^b^	2.8	2.5	−2.2 to 7.9	61.6	1.2	1.1	.270
*From 12 weeks to one-year follow-up*							
Time ^a^	−.83	.30	−1.4 to −.23	55.9	39.4	−2.8	.008
Time x treatment ^b^	−.91	.38	−1.7 to −.14	48.6	5.6	2.4	.022
**MADRS**							
*From pre-treatment to 12 weeks*							
Time ^a^	−9.5	15	−12.5 to −6.5	59.7	60.4	−6.3	.000
Time x treatment ^b^	1.9	1.8	−1.7 to 5.5	49.6	1.1	−1.0	.390
*From 12 weeks to one-year follow-up*							
Time ^a^	−.22	.25	−.72 to .29	49.6	15.8	−.87	.302
Time x treatment ^b^	−.91	.33	1.6 to .23	45.8	7.4	2.7	.009

*Note*: a. Change rate for the non-transference work group; b. Difference in change rates for the two treatment groups. For instance, during the second time period (from 12 weeks into treatment to one-year follow-up), BDI for the non-transference work group decreased by .83 points for every 10 weeks, and BDI for the transference work group had decreased by 1.74 points for every ten weeks (.83 + .91 points)

For BDI, the transference work group changed significantly during the first time period. Since the time*treatment interaction was not significant, it can be assumed that the non-transference work group also changed significantly from pre-treatment to 12 weeks (Table 3). For the second time period interaction was significant, which implies that the two groups had different change rates from 12 weeks to one-year follow-up. Inspection of the parameter estimates in Table 3 shows that the transference work group improved more than the non-transference work group during this time period (.91 points for every tenth week).

**Fig. 2:**
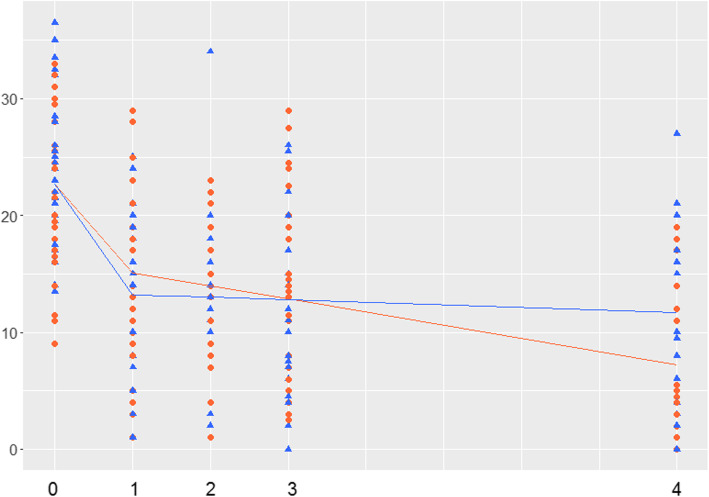
Mean trajectories for Montgomery and Åsberg Depression Rating Scale (MADRS) over time for patients in psychotherapy with or without transference interventions. The level of MADRS over time. 0, pre-treatment; 1 and 2, measures during therapy; 20 weeks; 3, post-treatment; 4, post-treatment

For MADRS, a similar picture was seen except that the non-transference work group did not change significantly from 12 weeks to one-year follow-up (Table 3). The transference work group improved significantly more than the non-transference work group (Fig. 2).

## Discussion

The present study had a dismantling design in which a single component in an existing and manualized treatment method (short-term psychoanalytic therapy) was varied. The efficacy of a specific technique (transference work) was investigated. On average, patients in both treatment groups showed a significant and large improvement measured with PFS, however no outcome differences between treatment groups. The level of depression decreased significantly more among the depressed adolescents in the transference work than the non-transference work group from mid-therapy (session 12) to 1 year post therapy when measured with the patient-rated BDI and the therapist- and evaluator rated MADRS. When the therapists encouraged adolescents to discuss their thoughts and feelings towards the therapist in the here and now (transference work), the young person improved significantly more on symptom measures.

The need for elaborating the patient-therapist relationship with adolescents in therapy is underlined [7, 21]. The treatment fidelity statistics show us that the dialogue focusing on the therapeutic relationship is present to a moderate extent in the transference work therapies, but absent from the non-transference therapy sessions, where this is not initiated by the therapist. Hence, adolescents, like the adults in the FEST study [22], do not by themselves discuss the therapeutic relationship or alliance, they need to be invited to do so. Then, the exploration of oneself in relation to the therapist, may comprise a balance between autonomy and acknowledgment, as well acceptance of oneself, which would be of importance for relieving depressive symptoms. Løvgren and colleagues [23] interviewed adolescents after transference work and non-transference work therapies, they found that the depressed adolescents reported the importance of the patient-therapist relation as helpful, characterised by an experience of confidence and trust in a supportive therapist. Could it be, that this experience is enhanced when the therapist invites the youngster to explore their feelings, including the negative ones, towards the therapist? Within psychodynamic theory, one aspect of depression is thought to be misplaced aggression directed inwards [12]. When the therapist invites the adolescent to criticize, express annoyance or frustration directed towards the therapist, it might assist to identify aggressive feelings that are not only directed towards self. This would be expected to reduce the depressive hold on the adolescent mind. According to Løvgren and colleagues, working with the relation to the therapist in the here and now also gave the adolescents an opportunity to explore the ongoing relationship, helping them to separate their inner world from outer reality something which is diluted while depressed, and to practice relational skills. Both may precede the reduction in depressive symptoms observed [23].

A central finding was that for depressive symptoms, differential change largely occurred at post-treatment. This effect seems also to be present for PFS but in a lesser degree and not significant at the alpha = .05 level. This non-significant finding might be due to a type II error, underlining the need for replication. Hundred patients were sought for the study, yet only 69 were included due to time limit and economic resources. In addition, not all adolescents came for follow up evaluations, 84% came for post-treatment evaluation and 64% attended one-year evaluation. We do not know if there are systematic variations in the group of adolescents that did not show, but the missing data analyses did not show any systemic variations (see Supplement). Only two patients received antidepressant medication. No adolescents from minority populations were referred to the study, and few boys were also referred, hence there are generalizability issues. The absence of a no-treatment control group restricts the assertion that the treatments were causally effective.

In their discussion of the IMPACT study, Asarnow and Ougrin [24] concluded that there was a need for more research aimed at “understanding key elements and mechanisms contributing to treatment effectiveness”. As an RCT investigating the effects of one single therapist technique, FEST-IT is in line with this research challenge. However, for therapists to be supported by empirical results when adjusting and tailoring their treatment approaches to the individual patient, more research on personalized treatment is needed. Still we do not know empirically why or for whom transference work is of significance in the depressed adolescent population. Increased knowledge is needed about what treatment works best for whom and what factors in the individual patient interact with the techniques of treatments, for better or for worse [25].

## Conclusion

There is a need to strengthen empirically support for which techniques are critical to improve the level of depressive symptoms in adolescents with MDD. Results from the main analyses in FEST-IT indicates that if the therapists for a specific age group of adolescent (16–18 years) focus on the patient-therapist relationship, explore the patient’s thoughts and feelings about the therapist, and negotiate the relationship, then this increases the treatment effects on depressive symptoms in the therapy.

## Supplementary Information

**Additional file 1.**

## Data Availability

The data set used in this article is available in anonymised version on request from the first author.

## Abbreviations

BDI: Beck Depression Inventory
CBT: Cognitive Behavioural Therapy
DSM-IV: Diagnostic and Statistical Manual of Mental Disorders, Fourth Edition
FEST: First Experimental Study of Transference–Interpretations
FEST–IT: First Experimental Study of Transference Work–In Teenagers
IMPACT: Improving mood with psychoanalytic and cognitive therapies”
MDD: Major Depressive Disorder
M.I.N.I.: Mini International Neuropsychiatric Interview
MADRS: Montgomery and Åsberg Depression Rating Scale
PFS: Psychodynamic Functioning Scales
SIDP-IV: Structured Interview for DSM-IV Personality
