# Supplementary material for: The first experimental study of transference work–in teenagers (FEST–IT): a multicentre, observer- and patient-blind, randomised controlled component study
Source: BMC Psychiatry. 2021 Feb 17;21:106. doi: 10.1186/s12888-021-03055-y (PMC7888176; doi:10.1186/s12888-021-03055-y)
Supplement: Supplementary file 1 — Additional file 1. [file 12888_2021_3055_MOESM1_ESM.docx]

**Supplementary appendix**

**Supplement to:** Ulberg R, Hummelen B, Hersoug AG, Midgley N, Høglend P, Johnsen H-S D: The First Experimental Study of Transference Work–In Teenagers (FEST–IT): a multicentre, observer- and patient-blind, randomised controlled component study

Appendix I: Planned interventions and summary of clinical protocol

Appendix II: Statistics

**Appendix I: Planned interventions and summary of clinical protocol**

FEST-IT was a multicentre, observer- and patient-blind, randomised controlled component study. The study had two treatment arms. Short Term Psychoanalytic Psychotherapy (STPP) with and without transference interventions.

**Short Term Psychoanalytic Psychotherapy (STPP)**

Psychoanalytic psychotherapy with youth is a well-established and empirical supported Empirical evidence supports treatment with psychoanalytic psychotherapy in adolescents (1) (2, 3). Short-term psychoanalytic psychotherapy has been shown to promote improvement in depressed young (4) *.*STPP is one of several psychological therapies recommended by NICE as equally effective in the acute treatment of child and adolescent depression. The therapy is based on general psychodynamic principles (e.g. attachment theory, developmental psychology, object relation theory, relational theory, and affect focus theory).

Therapists were experienced psychologists and psychiatrists. The therapists had a minimum of two years of formal clinicalpractical - theoretical psychodynamic/psychoanalytic training. As part of the preparation for being therapist in FEST-IT, the therapists were trained through a one-year course based on the treatment manual (5). During the preparation course, the focus was on the general psychodynamic principles emphasized in the treatment manual. In addition, training in the differences in the techniques when offering short-term psychoanalytic psychotherapy with or without transference work was included in the course. In the 1-year program therefor were trained to provide dynamic psychotherapy with a moderate frequency of transference interventions (one to three per session) and dynamic psychotherapy without transference work (6). To maintain the quality of the therapies and adherence to the manual, peer supervision in groups was offered throughout the study period. This also was to ensure that the therapy mode in each therapy group was delivered. Certified supervisors in psychoanalytic/psychodynamic psychotherapy managed the continuous training. The Principal Investigator and the study coordinator were available for the therapists at any time.

The treatment manual describes a 28-session treatment. In FEST-IT if the young person accepted, parents or carers were offered sessions with separate parent worker or with the therapist. These sessions were not part of what was investigated in the present study. The main purpose of psychotherapy with young people with depression is reduction of depressive symptoms. However, psychoanalytic psychotherapy also to aims foster dynamic change in the young person. Positive dynamic change includes a broad spectre of areas; i.e. improved relations, increased insight in one’s own reactions, improved tolerance for affects, and improved capacity to solve upcoming problems in life. STPP aims to help the adolescent to a healthy development socially; with peers and parents as well as prepare for the transition from adolescence to participation in the adult work force.

STPP with youth engage the young person in the search for understanding and insight in their own relationships, feelings, and background for the choices they make. The therapist’s role is to help the young person to understand more of his/her own unconsciousness. “This attentiveness to unconscious phenomena is specific to psychoanalytic psychotherapy, and is related to the theoretical importance attributed to these deeper less accessible layers of the mind.” (5). To help improve dynamic change, the therapists in dynamic/psychoanalytic psychotherapy uses transference interventions (TI). TI is thought to be a key ingredient in this kind of psychotherapy. However, whether it is a productive tool in therapy with depressed young people is unknown. In FEST-IT the therapists were instructed to use five categories of TIs in therapies with transference interventions (7, 8):

1) The therapist addressed transactions in the patient-therapist relationship (address transaction)

2) The therapist encouraged exploration of thoughts and feelings about the therapy and the therapist’s style and behavior (thoughts and feelings about therapy).

3) The therapist encouraged patients to discuss how they believed the therapist might feel or think about them (beliefs about therapist).

4) The therapist included him-/herself explicitly in interpretive linking of dynamic elements (conflicts), direct manifestations of transference, and allusions to the transference (linking therapist to dynamic).

5) The therapist interpreted repetitive interpersonal patterns (including genetic interpretations) and linked these patterns to transactions between the patient and the therapist (repetitive interpersonal pattern).

**Appendix II: Statistics**

*Modelling*

Supplemental table A gives the fit indices of the different models that were tested. The first step in the analyses aimed at modelling the mean structure of the data and addressed the question whether there was significant change over time for the entire sample. Though this answering this question is not the main aim of FEST-IT, it provides a platform for the construction and evaluation of subsequent models. This model included two covariates, the fixed intercept for the dependent variables and “time”, i.e., the length between each measurement occasion. “Time” was coded as integers and each integer represents approximately 10 weeks. For PFS, time was coded as 0-3-8 (baseline; post-treatment; one year follow-up). For BDI and MADRS, time was coded as 0-1-2-3- 8 (baseline; 12 weeks; 20 weeks; post-treatment; one-year follow-up. Three different covariance matrices were tested, i.e., diagonal, autoregressive type I, and unstructured. The model with unstructured covariance matrix gave significant better model fit than both the model with diagonal- and the model with autoregressive covariance matrix, for all measures. Therefore, the unstructured covariance matrix was used in the subsequent models.

The second step aimed at investigating whether there was differential change for the two treatment groups by including the interaction component “time*treatment”. As can be seen in table A, comparing model 1.1 and 2.1, model fit improved marginally by including this interaction. For PFS and BDI, the improvement of the model fit bordered at significance (*χ*^2^=3.6, p=.058 and *χ*^2^=3.5, p=.061, respectively) whereas improvement was significant for MADRS (*χ*^2^=5.0, p=.025).

The third step of the analyses addressed the question whether there were differences in change rates between treatment groups for different time periods. In these analyses, the fit of a pure linear model (a straight regression line through the different measurement occasions) was compared with a “linear spline model”, i.e., a model with a knot at one of the intermediate measurement occasions. This model implies that change trajectories were analyzed for two different time periods within the same model. The location of the knot was based on visual inspection of the scatterplots including interpolation lines. For PFS, the knot was placed at post-treatment (see supplemental figure 1), which implies that the first time period was from baseline to post-treatment and the second time period from post-treatment to one-year follow-up. This model includes two time variables, labeled S1 and S2; S1 was coded as 0-3-3 and S2 was coded as 0-0-5. See Fitzmaurice, Laird, & Ware (9) for details about coding of time variables. For BDI and MADRS, the knot was placed at the second measurement occasion. Thus, the first time period was from baseline to 12 weeks and the second time period from 12 weeks to one-year follow-up (supplement figure 2 and 3). S1 was coded as 0-1-1-1-1 and S2 was coded as 0-0-1-2-7.

As part of the sensitivity analyses, all steps were repeated using a competing model including a random intercept and random slope for the first time period and a variance component matrix for the covariances of the random effects (“random effects model”). The impact on model fit of including a random slope for the second time period (S2) was also tested. For PFS, the final Hessian matrix was not positively definite when including a random slope for S2; for BDI model fit did not improve significantly (*χ*^2^=2.8, df=1, p=.094, AIC went down with .8 points); for MADRS model fit deteriorated minimally and non-significantly (*χ*^2^=.5, df=1, p=.480, AIC went up with 1.5 points). Thus, only random slopes for S1 were included in the random effects models. As can be seen in table A, comparing the first and second sub-steps, i.e., x.1 versus x.2, the fit of the random effects model was almost always poorer than the original model, i.e., the model with the unstructured covariance matrix. The only exception was for PFS since there was no difference in fit between the spline model with random effects and the original linear spline model (step 3.1 versus step 3.2 in table A).

For all three steps, the F-values of the random effects model were of the same magnitude as the model with the unstructured covariance matrix. Moreover, none of the p-values lost their significance at the α =.05 level with one exception; in the simple linear random effects model for PFS, the p-value of the time*treatment interaction went up from .048 to p=.086. For BDI, the interaction component (time*treatment) had an F-value of 3.9 (p=.049) for the second time period in the linear spline model, and for MADRS, this value was 6.3 (p=.013). These values were 5.6 (p=.022) and 7.4 (p=.009) respectively in the main analyses (see table 3 in the paper).

*Missing data at baseline*

None of the patients had missing PFS at baseline. One patient had missing values for all MADRS scores at baseline, which were imputed by regressing BDI on MADRS. Six patients had missing values for all BDI scores at baseline, which were imputed by regressing MADRS on BDI. None of the patients had missing for both BDI and MADRS. The correlation between BDI and MADRS at baseline was .575, indicating that it is defensible to use BDI to impute MADRS and vice versa. Missing data at baseline were caused by a procedure failure and were therefore considered as completely missing at random.

*Missing PFS*

Sixteen patients had missing PFS at one occasion and 10 patients had missing PFS at two occasions. Eleven patients had missing PFS at post-treatment and 25 patients had missing at one-year follow-up. Chi square analyses showed that there were no significant differences in the prevalence of patients with missing PFS across the two treatment groups at these measurement occasions (*χ*^2^ = .65, p=.419 at post-treatment and *χ*^2^=.004, p=.947 at one-year follow-up).

A series of subsequent ANOVA analyses were performed to address the question whether patients with missing PFS were significantly different with respect to mean BDI and mean MADRS at the different measurement occasions. Mean values for four different groups were compared: 1) patients in transference treatment group without missing PFS at one-year follow-up; 2) patients in the transference group with missing PFS at one-year follow-up; 3) patients in non-transference group without missing PFS at one-year follow-up; and 4) patients in the non-transference group with missing PFS at one-year follow-up. ANOVA analyses including Tukey post hoc tests for mean BDI and mean MADRS were conducted for all measurement occasions except one-year follow-up. No significant differences emerged, not for the ANOVAs and neither for the post-hoc tests.

*Missing BDI*

With respect to missing BDI, 16 patients had missing BDI at one occasion; 12 had missing at two occasions; 9 had missing at three occasions; and 7 had missing at four occasions. Moreover, 6 patients had missing data at baseline; 22 patients had missing at 12 weeks; 33 had missing at 20 weeks; 11 had missing at post-treatment; and 23 had missing at one-year follow-up. A series of chi square analyses showed that there were no significant differences between the two treatment groups at these 5 measurement occasions with respect to the number of patients with missing BDI (largest chi square value = .559, p=.455).

A series of ANOVA analyses were conducted with Tukeys post-hoc tests in order to compare mean PFS across four groups: 1) patients in transference treatment group with complete BDI; 2) patients in the transference group with missing BDI; 3) patients in non-transference group with complete BDI; and 4) patients in the non-transference group with missing BDI. It should be noted that these groups were not identical at the different occasions. Thus, these analyses were performed four times; for missing BDI at 12 weeks; for missing BDI at 20 weeks; for missing BDI at post-treatment; and for missing BDI at one-year follow-up. No significant differences emerged.

*Missing MADRS*

9 patients had missing MADRS at one occasion; 11 patients had missing MADRS at two occasions; 9 patients had missing MADRS at three occasions; and another 9 patients had missing at four occasions. One patient had missing MADRS at baseline; 23 patients had missing MADRS at 12 weeks; 33 at 20 weeks; 11 at post-treatment; and 26 at one-year follow-up. Chi square analysis showed that there were no significant differences between the two treatment groups with respect to the number of patients with missing MADRS (smallest p-value =.513). The same types of ANOVAs were performed for MADRS as for PFS and BDI. No significant differences came to the fore.

Though it is not possible to give a definite answer to the question whether missing data were “missing at random” or “completely missing at random (10), the results of these analyses indicate that missing data could be considered as completely missing at random for the variables involved in the current study. Since the parameters and standard deviations were obtained by maximum likelihood estimation, missing data were therefore not imputed.

*Sensitivity analyses; outliers*

Patients with deviating response profiles were identified by visual inspection of scatterplots including individual interpolation lines. For PFS, no outliers were identified; for BDI one outlier was identified; and for MADRS two outliers were identified. Running the mixed models for BDI without this one patient did not result in any substantial differences. Notably, the interaction component for time*treatment was still significant (F=6.1, p=.017). Running the mixed models analyses for MADRS without the two outliers neither gave rise to any essential differences of the results, e.g., the interaction component between time*treatment kept its significance at the α =.05 level (F=6.0, p=.018).

*Sensitivity analyses; controlling for the possibility of baseline differences*

A third set of sensitivity analyses addressed the question whether there were significant differences between the two groups at baseline with respect to the three outcome variables. A series of independent sample t-tests showed that this was not the case (t=.14, p=.886 for PFS; t=.37, p=.714 for BDI; and t=1.64, p=.105 for MADRS). Accordingly, in the mixed models analyses, the “treatment” component at baseline was not significant for any of the measures (p= .885 for PFS; p=.513 for BDI; p=.081 for MADRS), and this component was therefore not included in the final analyses. Model fit did not improve by including this component, i.e., AIC went slightly up for PFS and BDI, and went negligibly down for MADRS (.8 points). However, for BDI, the p-value of the interaction between time*treatment became slightly larger than .05, i.e., .053.

|  | Psychodynamic Functioning Scale | | Nr* | Beck Depression Inventory | | Nr* | MADRS | | Nr* |
| --- | --- | --- | --- | --- | --- | --- | --- | --- | --- |
|  | LLH | AIC |  | LLH | AIC |  | LLH | AIC |  |
| Step 1.1 Linear model with unstructured covariance matrix  Step 1.2 Linear model with random intercept and random slope | 1039.7  1051.7 | 1055.7  1061.7 | 8  5 | 1840.2  1894.2 | 1874.2  1904.2 | 17  5 | 1673.1  1721.2 | 1707.1  1731.2 | 17  5 |
| Step 2.1 Linear model with interaction time*treatment; unstructured covariance matrix  Step 2.2 Linear model with interaction time*treatment; random intercept and random slope | 1036.1  1048.8 | 1054.1  1060.8 | 9  6 | 1836.7  1893.2 | 1872.7  1905.2 | 18  6 | 1668.1  1719.6 | 1704.1  1731.6 | 18  6 |
| Step 3.1 Linear spline model with time*treatment interaction for each time period; unstructured covariance matrix  Step 3.2 Linear spline model with time*treatment interaction for each time period, random intercept and random slope for S1 | 1024.6  1025.4 | 1046.6  1041.4 | 11  8 | 1814.6  1843.0 | 1854.6  1859.0 | 20  8 | 1638.1  1647.3 | 1678.1  1663.3 | 20  8 |

Note: Nr*= number of parameters. *Likelihood chi square tests PFS:* 1.1 versus 1.2 (*χ*^2^=18.0, df=3, p=.0004); 1.1 versus 2.1 (*χ*^2^=3.6, df=1, p=.058); 2.1 versus 2.2 (*χ*^2^=12.7, df=3, p=.005); 2.1 versus 3.1 (*χ*^2^=11.5, df=2, p=.003); 3.1 versus 3.2 (*χ*^2^=1.2, df=3, p=.753). *Likelihood chi square tests BDI:* 1.1 versus 1.2 (*χ*^2^=54.0, df=12, p<.00001); 1.1 versus 2.1 (*χ*^2^=3.5, df=1, p=.061); 2.1 versus 2.2 (*χ*^2^=56.5, df=12, p<.00001); 2.1 versus 3.1 (*χ*^2^=22.1, df=2, p=.00006); 3.1 versus 3.2 (*χ*^2^=28.4, df=3, p<.00001); *Likelihood chi square tests MADRS:* 1.1 versus 1.2 (*χ*^2^=48.1, df=12, p<.00001); 1.1 versus 2.1 (*χ*^2^=5.0, df=1, p=.025); 2.1 versus 2.2 (*χ*^2^=51.5, df=12, p<.00001); 2.1 versus 3.1 (*χ*^2^=22.1, df=2, p=.00006); 3.1 versus 3.2 (*χ*^2^=26.0, df=3, p<.00001).

***Table A:* Fit indices**


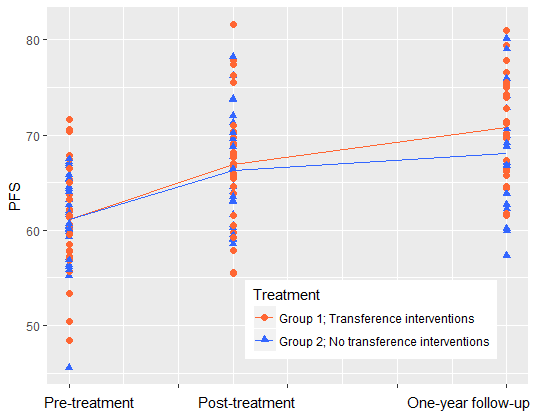


***Figure 1:*** Mean trajectories for Psychodynamic Functioning Scales (PFS) for patients in psychoanalytic psychotherapy with or without transference intervention.


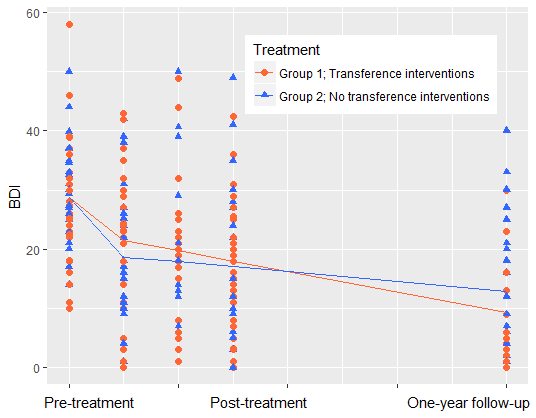


***Figure 2:*** Mean trajectories for Beck Depression Inventory for adolescents in psychoanalytic psychotherapy with or without transference intervention

**References**

1. Midgley N, O’Keeffe S, French L, Kennedy E. Psychodynamic psychotherapy for children and adolescents: an updated narrative review of the evidence base. Journal of Child Psychotherapy. 2017:1-23.

2. O’Keeffe S, Martin P, Goodyer IM, Wilkinson P, Consortium I, Midgley N. Predicting dropout in adolescents receiving therapy for depression. Psychotherapy Research. 2017:1-14.

3. Goodyer IM, Reynolds S, Barrett B, Byford S, Dubicka B, Hill J, et al. Cognitive behavioural therapy and short-term psychoanalytical psychotherapy versus a brief psychosocial intervention in adolescents with unipolar major depressive disorder (IMPACT): a multicentre, pragmatic, observer-blind, randomised controlled superiority trial. The lancet Psychiatry. 2016.

4. Trowell J, Joffe I, Campbell J, Clemente C, Almqvist F, Soininen M, et al. Childhood depression: a place for psychotherapy. An outcome study comparing individual psychodynamic psychotherapy and family therapy. Eur Child Adolesc Psychiatry. 2007;16(3):157-67.

5. Cregeen S, Hughes C, Midgley N, Rhode M, Rustin M. Short-term psychoanalytic psychotherapy for adolescents with depression: A treatment manual: Karnac Books; 2016.

6. Ulberg R, Hersoug AG, Hoglend P. Treatment of adolescents with depression: the effect of transference interventions in a randomized controlled study of dynamic psychotherapy. Trials. 2012;13:159.

7. Hoglend P, Bogwald KP, Amlo S, Marble A, Ulberg R, Sjaastad MC, et al. Transference interpretations in dynamic psychotherapy: do they really yield sustained effects? Am J Psychiatry. 2008;165(6):763-71.

8. Ulberg R, Amlo S, Hoglend P. Manual for Transference Work Scale; a micro-analytical tool for therapy process analyses. BMC Psychiatry. 2014;14:291.

9. Fitzmaurice GM, Laird NM, Ware JH, editors. Aplied longitudinal data analysis, second edition: John Wiley & Sons; 2004.

10. Enders CK. Applied missing data analysis: Guilford press; 2010.
